# Choropleth Map Design for Cancer Incidence, Part 2

**Published:** 2009-12-15

**Authors:** Thomas B. Richards, Zahava Berkowitz, Cheryll C. Thomas, Stephanie Lee Foster, Annette Gardner, Jessica Blythe King, Karen Ledford, Janet Royalty

**Affiliations:** Centers for Disease Control and Prevention; Centers for Disease Control and Prevention, Atlanta, Georgia; Centers for Disease Control and Prevention, Atlanta, Georgia; Centers for Disease Control and Prevention, Atlanta, Georgia. Stephanie Lee Foster is also affiliated with the Agency for Toxic Substances and Disease Registry, Atlanta, Georgia; Centers for Disease Control and Prevention, Atlanta, Georgia; Centers for Disease Control and Prevention, Atlanta, Georgia; Centers for Disease Control and Prevention, Atlanta, Georgia; Centers for Disease Control and Prevention, Atlanta, Georgia

## Abstract

Choropleth maps are commonly used in cancer reports and community discussions about cancer rates. Cancer registries increasingly use geographic information system techniques. The Centers for Disease Control and Prevention's Division of Cancer Prevention and Control convened a Map Work Group to help guide application of geographic information system mapping techniques and to promote choropleth mapping of data from central cancer registries supported by the National Program of Cancer Registries, especially for comprehensive cancer control planning and evaluation purposes. In this 2-part series, we answer frequently asked questions about choropleth map design to display cancer incidence data. We recommend that future initiatives consider more advanced mapping, spatial analysis, and spatial statistics techniques and include usability testing with representatives of state and local programs and other cancer prevention partners.

## Introduction

In part 1 of this 2-part series, we answered frequently asked questions about the purpose of choropleth maps, geographic units of analysis, cancer sites, age-adjusted rates, rate ratios, and reliability. In Part 2, we address suppression rules to protect the privacy and confidentiality of cancer patients; questions related to mapping cancer stages, rates, and percentages; classes for map display; comparing maps over time; map color schemes, labels, projections, and output media; and limitations in interpretation (1).

## Frequently Asked Questions About Choropleth Map Design

### 1. *Are suppression rules designed for table cells also appropriate for maps?*

To protect the privacy and confidentiality of people with cancer, *United States Cancer Statistics* reports require cells in tables to be suppressed when the rates are based on 15 or fewer cases (2).

Application of data suppression rules for table cells also should be considered when developing cancer incidence choropleth maps. Data suppression rules are especially appropriate when counts are displayed. However, the suppression rules may be overly restrictive when rates are classified categorically or when the rate for an index county is calculated as the combined average of rates in the index county and its contiguous neighboring counties.

### 2. *Should maps show advanced cancer stage, localized stage, or both?*

A tenet of cancer screening is to diagnose cancer before symptoms develop, when cancer is at a less advanced stage and more favorable treatment outcomes are possible (3). A map series displaying stage at diagnosis over time for cancers with effective screening modalities may be useful as part of efforts to assess gaps in cancer prevention and control (4). Maps of advanced stage, localized stage, or both can be useful in the context of cancer prevention and control planning and evaluation. For example, identifying areas with high incidence of advanced-stage colorectal cancer would suggest the need for evaluation to determine how cancer screening efforts may be improved (5). Conversely, identifying areas with more cases of localized than advanced-stage cancers could be useful as part of efforts to monitor whether early detection strategies were successful.

### 3. *Should maps show rates by stage, percentage of advanced-stage to total cases, or both?*

Both maps of advanced-stage rates and maps of the proportion of advanced-stage cases can be useful, but they answer different questions. Maps of advanced-stage breast cancer rates answer questions about the distribution of advanced-stage cancer in relation to the population at risk. In contrast, maps of the proportion of advanced-stage breast cancer cases depend on the relative number of advanced- and localized-stage cases, and provide insights into that relationship.

Both rates and percentages use the same numerator (eg, the number of advanced-stage breast cancer cases). However, in rates, the denominator is the study population at risk for developing the disease. In contrast, with percentages, the denominator consists of the total cases across all categories of cases (eg, the total of all stages of breast cancer).

The spatial patterns observed on rate maps may not be the same as the patterns observed on maps of percentages. For example, suppose that a county has a relatively large number of women at risk for breast cancer, the total number of cancer cases is small, but a large proportion of those cases are advanced stage. The rate map would display the county as having a low advanced-stage breast cancer incidence rate, whereas a map of percentages would display the same county as having a high proportion of advanced-stage breast cancer cases.

### 4. *What method should be used for grouping age-adjusted cancer incidence rates into categories for map display?*

The choice of categories for grouping rates for mapping can affect the visual appearance of the map and thereby interpretation of mapped results. Selecting the interval may depend on the purpose of the map, the underlying data distribution, and the intended audience for the map (6-8). Map legends automatically generated by geographic information system (GIS) software programs typically provide information about the range in rate values for each rate category along with the color assigned to that category. Adding details on the number of geographic units included in each class interval may help map readers understand the distribution of the information mapped. The counts for each category can be manually added next to each category on the map legend or displayed in a frequency distribution graph included on the map.

Four examples of classification methods (6) are the following:

- *Quantiles:* The rates for the areas of interest (eg, counties) are first rank-ordered, and then an equal number of observations are placed in each class. The number of classes determines the specific type of quantile map (eg, 3 classes are referred to as tertiles, 4 classes as quartiles, and 5 classes as quintiles). Quantile maps can be helpful in identifying the spatial patterns of the relative rankings of rates within the geographic units of interest (eg, counties).
- *Equal intervals:* The range of the entire dataset (ie, the difference between the upper and lower values for the county age-adjusted incidence rates within a state) is divided by the desired number of data classes. The upper and lower values for the classes are assigned so that each class has the same width interval (distance between the upper and lower values for that class) without regard to the number of geographic units (eg, counties) included in each class. Equal interval maps can be used to help identify spatial patterns of absolute values of rates and locations of extreme outliers.
- *Natural breaks (also known as optimal breaks or Jenks' method):* An iterative algorithm is applied to define classes where the variance is minimized within all classes and maximized among classes.
- *Standard deviations:* Data are assigned to classes on the basis of where they fall relative to the mean and standard deviations of the data distribution. This classification method may be used to help identify and contrast geographic units (eg, counties) that are above and below the mean.

Brewer (6,8) reports that epidemiologists tend to prefer quantile maps for age-adjusted incidence rates. In selecting the number of quantiles, the map designer needs to balance several conflicting considerations. On the one hand, a larger number of quantiles may be advantageous because, as the number of quantiles increases, the observed spatial patterns become more stable. On the other hand, as the number of quantiles increases to 7 or more, it may be increasingly difficult for map readers to distinguish between the colors assigned to the different classes on the map legend (6).

### 5. *What classes should be used to group data when the goal is to compare a series of maps over time?*

If the goal is to compare changes between maps over 2 or more time periods, each rate map in the time series should be prepared by using the same set of intervals to categorize rates (6,9). To identify optimum intervals for this type of map series, the rate data for all time periods is typically pooled. Appropriate rate categories are defined by using the combined data, and then maps are developed for each time period by using those rate categories.

However, if the goal is to identify areas with the highest and lowest rates on each map in the time series, then intervals that distinguish high and low rates should be defined separately for each map, on the basis of rate data to be displayed in each map.

### 6. *How should map color schemes be selected so that maps can be interpreted by color-blind map readers?*

Some color schemes (eg, red-green) can result in maps that are difficult for color-blind people to read (6). *VisCheck* is a Web-based tool that people with normal color vision can use to get a sense of how color-blind people see colors (10).

Colors for maps should be selected to be consistent with Section 508 of the US Rehabilitation Act, which requires federal agencies to make their electronic and information technology accessible to people with disabilities (11). *ColorBrewer* (Penn State, University Park, Pennsylvania), a Web-based tool that helps identify and create good color schemes for maps and other graphics, indicates which recommended color schemes work well for people with red-green color blindness (12,13). For users of ArcGIS software, the National Cancer Institute has developed an ArcGIS extension known as *ColorTools*, which facilitates the preparation of GIS maps using the color schemes recommended in *ColorBrewer* (12,14).

### 7. *What labels should be included on a map?*

Map labeling may vary depending on the study question and audience. Geographic text labels (eg, state and county names) can be used to identify specific areas of interest. The number and size of text labels may need to be reduced so that users can identify spatial patterns. As an alternative to detailed text labels, geographic clues (eg, point locations of major cities, lines for highways, and locations of rivers and lakes) can be used to help orient community members or decision makers. One advantage of Web-based maps is the ability to hide text labels until the user passes a mouse cursor over an area (15,16).

### 8. *What map projection should be used?*

Geographical coordinate systems enable locations to be identified on a 3-dimensional spherical surface (17). Basic building blocks for a geographical coordinate system include a *datum* (a reference point for measuring the origin and orientation of latitude and longitude lines); latitudes and longitudes (angles measured from the center of the sphere to reference points on the sphere's surface); and a prime meridian (a reference point for other longitudes).

The actual surface of the earth is more complex than a sphere. Spheroids (defined by rotation of an ellipse with a longer and shorter axis) provide more accurate representation (17). Different areas on the earth's surface may require a specific spheroid to best fit a specific location, and the best spheroid for 1 region is not necessarily the best for another. A variety of projected coordinate systems have been proposed to best represent spheroid surface locations on a flat, 2-dimensional surface (eg, a sheet of paper).

Every map projection distorts distance, area, shape, direction, or some combination of those factors. Choosing the most appropriate projection depends on the study question. Also, agencies or organizations may prefer a specific projection standard.

The Figure compares a US map that uses unprojected geographic coordinates with a map that uses the Albers Equal Area Conic projected coordinates. The Albers Equal Area Conic projection is commonly used for thematic maps showing large areas of the United States that are mainly east-west in extent (eg, maps of the 48 contiguous states). This projection is *equal-area* because area is not distorted (17).

**Figure.**
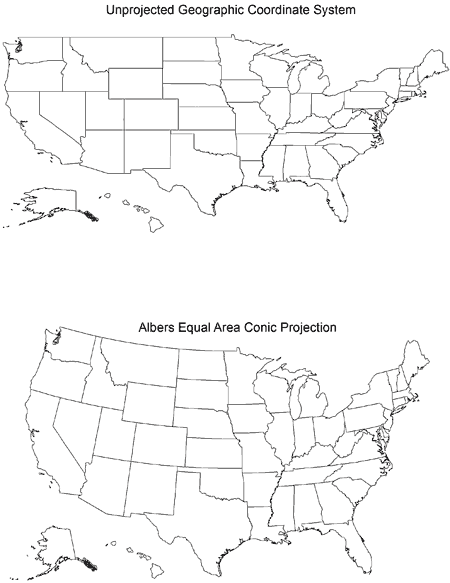
Two drawings of a US map. The top map uses unprojected geographic coordinates; the bottom map uses the Albers Equal Area Conic projected coordinates.

Other projections are used for maps at the state or county level (17). Two examples are the Universal Transverse Mercator projections, which divides the 48 contiguous US states into 10 zones (each of which has a specific projection) and the State Plane Coordinate System projections, which divides the 48 contiguous US states into 110 zones (each of which has a specific projection).

### 9. Will the same map work for all output media?

When a map is being developed, consider how it will be used or published. Color maps are usually used for PowerPoint presentations; either color or black-and-white maps (including shades of gray) may be used for publication in a scientific journal. Although the starting point for each of these maps is the same information, changing the map from 1 medium to another may require significant changes in the map design.

Colors should be checked for each output media. For example, if the goal is a color map for a PowerPoint presentation, RGB (red-green-blue) colors would be optimal, but if the goal is publication of a hard copy, high-quality color map, CMYK (cyan-magenta-yellow-black) colors may be a better choice. Map colors may have a different appearance when displayed on a computer monitor, a projector, or a color printer. The *ColorBrewer* Web site includes guidance on the usability of different color schemes for a variety of uses (eg, desktop computer monitors, laptop computers, room projectors, color printers, photocopiers, color-blind users) (12).

Similarly, to successfully print a black-and-white copy from a color map, the classes for rates may need to be limited to 4 or 5 categories so that shades of gray can be distinguished. Alternatively, patterns such as hatch marks or other fill symbols and labels may need to be added to help map readers distinguish categories.

### 10. *What limitations can affect interpretation of choropleth cancer incidence maps?*

When choropleth maps are presented, comprehensive cancer control decision makers should be reminded of the following constraints on interpretation:

- Administrative-political boundaries reflect administrative-political needs and may have little relationship to the true spatial distribution of cancer incidence.
- If a series of maps is prepared by using geographic units of analysis with different shape and area, the appearance of observed spatial patterns may appear to change. Geographers refer to this phenomenon as the *modifiable areal unit problem* (18).
- Because choropleth maps fill the geographic unit of interest with the color corresponding to that unit's rate, community decision makers may incorrectly assume that cancer rates are constant across the entire unit (19). For example, suppose the map shows cancer rates by county. Many US counties include neighborhoods with diverse characteristics (20). Consequently, for neighborhood planning, maps at the community level that apply more advanced mapping methods may be needed (19).
- Choropleth maps use geographic units of analysis to aggregate individual case data. Inexperienced map readers may want to draw conclusions about cause-and-effect relationships from maps. However, relationships suggested by aggregated data may not be the same as those at the individual level (21). Epidemiologists refer to this phenomenon as the *ecological fallacy* (18).
- Population size may vary considerably among the geographic units displayed on a map. Geographic units with small populations (eg, rural counties) may have rates that are extremely high or low because of small-number problems (19).
- Caution also is needed regarding interpretation of the spatial patterns close to the edges of the study area (18). For example, when a map displays cancer rates by county in a single state, decision makers can consider the rates in all directions around a county of interest located toward the center of a state. However, if the county of interest is located along the state boundary, decision makers can only consider the rates in adjacent counties toward the center of the study state, because no information is displayed about rates in neighboring counties outside the state boundaries. Geographers refer to this problem as *edge effects* (18).
- Application of methods that include information about rates in counties in contiguous states can help inform analysis of the spatial patterns along state boundaries. For example, in 2006, Gregorio et al (22) reported on spatial scan statistic cluster analyses in 3 states (Connecticut, Massachusetts, and Rhode Island) by using different size study areas. In 1 set of analyses, each state was considered as an independent, geographic unit. In other analyses, the data from all 3 states were combined and analyzed as a single geographic unit. The authors found that the individual state analyses provided different results than the combined state analyses (22). They concluded that spatial analysis results need to be considered conditional on the geographic area selected for study and recommended that efforts be made to maximize potential for data from different states to be pooled for combined state analysis.

## Conclusions

Some of the questions commonly raised about using GIS and mapping techniques to present cancer incidence data are covered in this article. However, the process of preparing responses to these questions was not straightforward, and our discussions raised additional issues for future consideration. More advanced mapping, spatial analysis, and spatial statistics techniques also have both strengths and limitations (23-25). Recommendations regarding more advanced methods should be considered as program needs dictate. Usability testing with representatives of state and local programs and other cancer prevention partners should be included as part of future initiatives in this area.
